# Mandatory Stent Removal for Eradication of Infection in a Hemodialysis-Dependent Patient: A Case Report

**DOI:** 10.7759/cureus.101441

**Published:** 2026-01-13

**Authors:** Khalid A Albrekeit, Mohamed A Albugami, Norah S Al Towaim, Bayan Albugami, Faisal A Albaqami

**Affiliations:** 1 Vascular Surgery, Prince Sultan Military Medical City, Riyadh, SAU; 2 College of Medicine, King Saud Bin Abdulaziz University for Health Sciences, Riyadh, SAU; 3 Medicine and Surgery, Imam Mohammad Ibn Saud Islamic University, Riyadh, SAU; 4 College of Medicine, Imam Mohammad Ibn Saud Islamic University, Riyadh, SAU

**Keywords:** balloon angioplasty with stent, end-stage renal disease (esrd), hemodialysis access, subclavian venous access, vascular occlusion

## Abstract

Venous stent infection is a rare but serious complication in hemodialysis patients, associated with significant morbidity. Early recognition and management remain challenging due to the nonspecific presentation. A 29-year-old female on maintenance hemodialysis presented with fever and leukocytosis following cephalic arch venoplasty and stent placement. Despite targeted intravenous antibiotics, she failed to improve clinically. Subsequent stent removal was performed, leading to resolution of the infection. This case underscores the importance of considering stent infection in febrile dialysis patients with recent venous intervention. It highlights that in immunocompromised hosts, prompt stent removal may be necessary for source control when medical therapy fails. This report adds to the limited literature on the management of infected hemodialysis access stents.

## Introduction

The preferred method for durable hemodialysis access is the creation of a native arteriovenous fistula, as it is associated with the highest rates of long-term patency and the lowest incidence of complications [1]. In upper arm fistulae, cephalic arch stenosis (CAS) is the leading cause of dysfunction and failure, accounting for 30-50% of dysfunctions/failures in these fistulae [2]. The cephalic arch’s anatomical configuration, in contrast to the otherwise ideal conduit of the upper arm cephalic vein, confers a significant risk for stenosis. This pathology directly precipitates the hemodynamic alterations and functional decline of the access [3]. Stent grafts represent the most durable endovascular option for CAS [4], significantly outperforming both venoplasty and bare-metal stents in primary and assisted patency, with evidence supporting their role as a primary treatment modality [5]. While stent deployment is generally feasible, procedure-related complications such as thrombosis, stent migration, and in-stent restenosis may arise [6]. Full endothelialization of metallic stents occurs around four weeks [7]. Infections reported in both arterial and venous territories are attributed to bacterial seeding during this pre-endothelialized period [8]. Infection of venous stents in hemodialysis patients remains a rare but serious complication with limited literature guiding optimal management. This case aims to alert clinicians to consider stent infection in febrile patients following venous stent placement, especially when bacteremia persists despite appropriate antibiotics. Furthermore, it contributes to the sparse evidence on the decision-making between conservative medical therapy and stent removal in immunocompromised hosts, aiming to inform future clinical practice in similar high-risk scenarios.

## Case presentation

**Figure 1:**
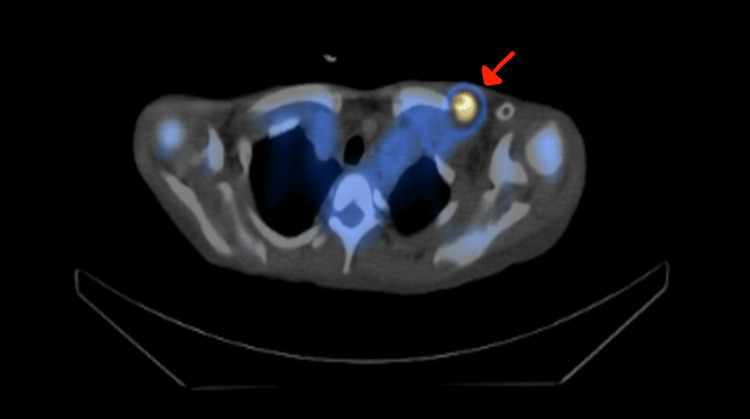
Whole-body nuclear medicine showing a localized focal area of increased radiolabeled white blood cell (arrowed) activity closely related to the vascular stent.

**Figure 2:**
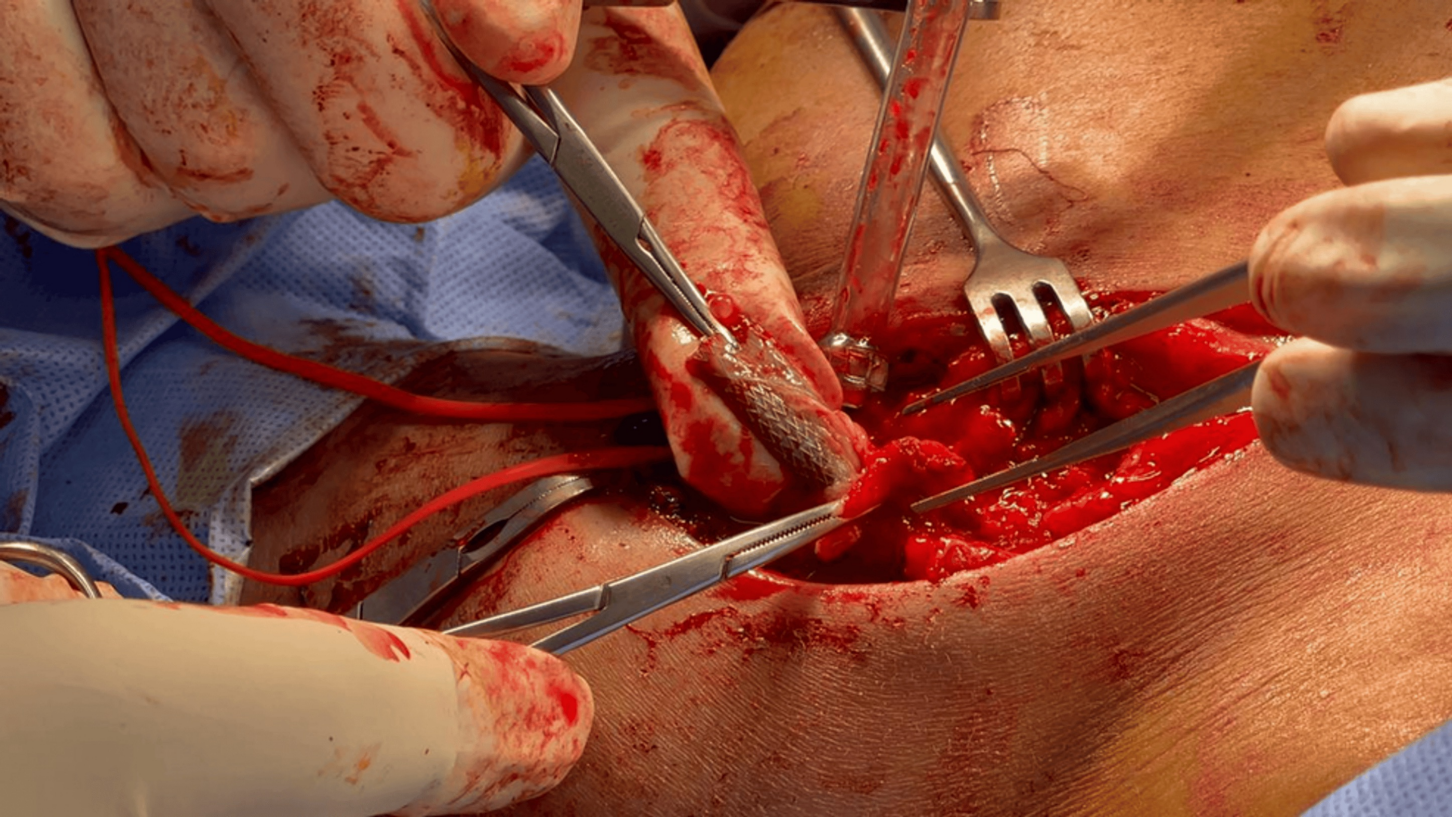
Infected stent removal via a superior incision.

A 29-year-old female, a known case of end-stage renal disease on regular hemodialysis through a left brachiocephalic fistula, was referred to our medical city with a four-day history of undocumented fever associated with chills in June 2022. The patient had a history of recent cephalic venous arch venoplasty and stent placement. The patient’s initial leukocyte count was 6,630 cells/mm³, C-reactive protein was 43 mg/dLm and blood culture showed *Pseudomonas aeruginosa*. Whole-body nuclear medicine testing showed a localized focal area of increased radiolabeled white blood cell (WBC) activity just below the lateral portion of the left clavicle, closely related to the medial superior end of the vascular stent (Figure 1). A CT scan showed no collection. The patient was managed conservatively and started on targeted intravenous antibiotic (ceftazidime) and improved clinically. She was discharged to outpatient antimicrobial therapy to complete a full six-week course; however, she presented one week later with a history of recurrent vomiting, loss of appetite, and high-grade fever with left upper arm swelling and tenderness and leukocytosis of 11,550 cells/mm³ and C-reactive protein of 202 mg/dL. The patient was readmitted, and antibiotics were upgraded to meropenem. The patient was prepared for surgical exploration of the infected graft and reconstruction of the fistula. Intraoperatively, there was a collection of pus as well as partial thrombosis of the subclavian vein; hence, the fistula was ligated, and the stent was removed via a superior incision (Figure 2). The patient tolerated the procedure well, and the wound was kept open for secondary closure. The patient improved clinically; her fever subsided, and inflammatory markers improved. She was discharged home in good condition. On follow-up in the clinic, the wound had healed, and the blood culture was negative.

## Discussion

We presented a case of a Gram-negative bacterial infection involving a recently deployed endovascular stent for CAS in a hemodialysis patient. This required definitive surgical removal after failed conservative management. CAS is a recognized and challenging complication in this population, arising from a multifactorial pathophysiology involving anatomical predisposition, hemodynamic stress from the arteriovenous fistula, and a resultant pathological healing response of intimal hyperplasia [9,10]. For such stenoses, endovascular stenting is often employed when surgical revision is prohibitive and angioplasty fails due to elastic recoil. Our case shifts focus to the critical, though rare, complication of infection within this therapeutic paradigm.

The diagnosis in our patient was confirmed by positive blood cultures, local signs, and a markedly positive indium-labeled white blood cell scan. Despite initiation of guideline-directed intravenous antibiotics, the persistence of fever and elevated inflammatory markers led a multidisciplinary team to conclude that the infected stent, as a retained foreign body, would preclude definitive cure with medical management alone, necessitating explantation for source control. This outcome aligns with the pathophysiological understanding that stent infection is a risk primarily before complete endothelialization, a process that typically takes several weeks [11,12]. The porous metallic scaffold of a stent provides a temporary nidus for bacterial seeding, a risk compounded in hemodialysis patients by frequent cannulation, an immunocompromised state, and the stent’s location within the access circuit [13].

This case underscores several key clinical principles. First, stent infection, though uncommon (<1%), must be included in the differential diagnosis for any patient presenting with fever, bacteremia, or local inflammation following recent stent placement [13]. Second, while stent salvage with prolonged antibiotics may be attempted in selected, stable patients, surgical removal remains the definitive therapeutic cornerstone when infection is established, as conservative management alone often fails [14]. Finally, this case reinforces that stent placement should be a judicious, last-resort option for access salvage. A high index of suspicion and early multidisciplinary planning are essential in managing this serious complication. Consideration of prophylactic antibiotics in the early post-placement period may be warranted for stents in high-risk locations, such as downstream from active cannulation sites.

## Conclusions

In this immunocompromised hemodialysis patient, conservative management with intravenous antibiotics failed to resolve the infection of a cephalic venous stent, necessitating definitive surgical removal for source control. This outcome underscores that in immunosuppressed individuals with device-related bacteremia, earlier intervention for stent explantation should be considered when there is a lack of clinical response to antimicrobial therapy.
